# Visual Analysis of International Environmental Security Management Research (1997–2021) Based on VOSviewer and CiteSpace

**DOI:** 10.3390/ijerph20032601

**Published:** 2023-01-31

**Authors:** Haizhen Cao, Hongxiang Ou, Weiyi Ju, Mengli Pan, Honglai Xue, Fang Zhu

**Affiliations:** School of Safety Science and Engineering, Changzhou University, Changzhou 213164, China

**Keywords:** environmental safety management, VOSviewer, CiteSpace, visual analysis of knowledge graph

## Abstract

To investigate the international development status and hot trends in the field of environmental security management in recent years, the published the environmental security management literature from 1997 to 2021, which was retrieved from Web of Science, with VOSviewer as the main and CiteSpace as the auxiliary, through the cooperation network of authors, scientific research institutions, and countries. The keywords were visualized by clustering, time zone analysis, and burst analysis. A total of 7596 articles were retrieved, forming six main clustering labels, including 28,144 authors. The research hotspots are from the fields of personal health, society, agriculture, ecological environment, energy, and sustainable development, as well as the development of internet environmental safety management, such as big data, Bayesian networks, and conceptual frameworks. Through cluster analysis, the cooperation of major research teams and scientific research institutions and the cooperation and development between countries were analyzed. The cooperation between scientific research institutions in various countries is relatively close. The United States currently occupies a dominant and authoritative position in this field. China has cooperated more closely with the United States, Britain, Australia, and India.

## 1. Introduction

Environmental safety can be expressed as a measure of the degree of harmony between man and the natural environment, which is based on the material basis of human adaptation to the living environment. The struggle between people and environmental safety disasters has lasted for a long time, and it has been occuring throughout the history of human development. Natural disasters [1] caused by natural causes, such as astronomical geography, weather, and hydrology, as well as soil biology, have seriously affected people’s safety. In addition, man-made disasters induced by humans’ own unsafe behavior have also contributed a malignant impact on environmental security. Environmental hazards, wars, nuclear threats, biosafety, and other issues are causing damage to environmental security, which is almost equivalent to natural disasters.

Environmental safety mainly includes production technology and social–political environmental safety, including urban public safety [2], mine safety [3,4] and factory workshop environmental safety. Environmental safety management generally requires professional safety researchers to carry out risk assessment [5,6,7] and hazard source investigation [8] on their production or living environment, so as to change the environmental space with potential safety hazards from danger level to safety level. Through the investigation of global coal mining problems, Chu [9] found that the main problems are divided into three categories: frequent accidents, occupational diseases, and environmental impacts. In their research, the mine safety and environmental protection of relevant coal producing countries are compared horizontally and vertically. It is considered that the environmental safety risk assessment of coal mines is an important link to protect workers and the environment. In order to reduce the safety and environmental problems of coal mines in the future, suggestions on prevention and control of water inrush and gas disasters are put forward. In order to understand the frontier development trend in the field of building environment safety, Zhou et al. [10] analyzed the previous studies from the perspective of time sequence and theme and found that there are four main research results: construction safety research perspective, research trend, innovative technology application, and safety information flow. These research results can provide effective guidance for future construction safety research. Asah-Kissiedu et al. [11] believe that safety, health, and environment management have become a priority event for enterprise construction units in reducing accidents and other negative effects and meeting the requirements of laws and regulations. By drawing on the concept of capability maturity model integration, a maturity model integrating safety, health, and environmental management is established. Dyrdonova et al. [12] proposed that environmental security is based on the concept of sustainable development. By using the systematic economic analysis method, the characteristics of the green industrialization process were studied, and the level of environmental expenditure was evaluated. An index system-monitoring conceptual model that allows the diagnosis of product greening technical parameter changes and the estimation of economic effects in the cost-effectiveness dimension is proposed. Sepulveda [13] summarized the security risk sources of software security systems, social technology models, and decision support softwares. It was found that there is currently no regulatory safety standard for decision support software, and professional and environmental professionals can bridge this defect by applying the safety management system process to these digital tools. Sharma et al. [14] analyzed 289 articles on occupational health and safety from 1991 to 2018 in the core collection of Web of Science with Bib Excel and HistCite software to understand the future trend and connection of their research. A time network of cross-domain trend evolution is proposed to link performance and the environment, and, in the future, environmental safety could be an integral part of more efficient assessment of safety performance and occupational safety research.

The quantitative analysis method based on mathematical statistics is called bibliometrics [15,16]. Through bibliometrics, the huge text information can be effectively sorted out with regards to time context of its development and the hot trend of future research. VOSviewer and CiteSpace software are two bibliometric softwares. They take the literature, keywords, authors, cooperative institutions, and countries as the main research objects, and they use mathematical statistics and other research methods to quantitatively analyze them. At present, international researchers have developed some bibliometric analysis softwares for drawing scientific knowledge maps, such as VOSviewer, Bib liometrix, CiteSpace, the Network Workbench (NWB), Pajek, Sci2, and Metaknowledge, which are currently suitable for topic analysis and network analysis. Compared with other bibliometric analysis softwares, VOSviewer has a stronger visualization effect. Moreover, the analysis function of this software is more comprehensive. In addition, one of the important features of CiteSpace is that keyword time zone analysis can be performed. This feature can more directly grasp the hot trend changes. Therefore, this study chooses VOSviewer and CiteSpace to visually analyze the field of environmental safety management to explore the field clustering and hot trends.

Researchers have applied VOSviewer and CiteSpace software in different research fields. For example, in the era of novel coronavirus pneumonia, VOSviewer software was used by Yu et al. [17] to analyze the rules of the medical literature and the current research status. Disease management, clinical features, and pathogenesis are the main directions of publication analyzed by the software. Zhang et al. [18] applied VOSviewer software to conduct a quantitative analysis of the research on spinal cord injury. Based on the visual analysis of a large number of studies, the future hot directions were summarized in their research. Chen et al. [19] used VOSviewer software to conduct a quantitative analysis of the literature on depression and breast cancer patients. Ten years of data on Web of Science have been analyzed in their research, so that the hotspots and frontiers of breast cancer and depression research have been identified. Sood et al. [20] applied VOSviewer software to conduct a scientific quantitative analysis of the literature in the field of vehicular communication. This research has accelerated the scientific research progress in the field of vehicle communication, and a new research method has emerged. Kuzior et al. used VOSviewer software to visually analyze blockchain technology [21] and studied the statistical literature by the clustering method. In their study, three main six-clusters were proposed. Li et al. [22] applied VOSviewer software to analyze the field of agricultural pollution. Based on the analysis of 1338 agricultural pollution data, the hot direction of environmental pollution has received extensive attention in their research. VOSviewer software was used by Li et al. [23] to analyze scientific research in the field of urban microclimate. Huang et al. [24] explored the knowledge map of the theme of resilient cities or communities from 1995 to 2022 through VOSviewer. The results showed that its development process was mainly divided into three periods: inattention, start-up, and rapid growth. Keyword analysis pointed out hot topics in different periods. In addition, many scholars have applied VOSveiwer software to conduct quantitative analysis of the literature in various fields to find popular research directions in the future [25,26,27]. Xiao [28] used CiteSpace software to visually analyze the literature on organic photovoltaic technology in the past decade of 2017 and identified the output and cooperation, research hotspots, important references, and future trends of organic photovoltaic technology. Li analyzed and sorted out all relevant matrices supported by CiteSpace for the information security risk (ISR) literature in the Web of Science, IEEE, ACM, and Scopus databases, discovered future research trends in ISR, and proposed an integrated visualization analysis based on knowledge and innovation in the ISR domain [29]. At present, scholars in the computer field have also applied it. For example, Wang et al. [30] studied the data related to big data in the Web of Science and CNKI databases from 2008 to 2017, clarifying the key research directions, the key literature, and hot frontiers in the field of big data research, predicting the future development trends of the field, and then they compared the research situation at home and abroad in order to provide some reference and help for readers and other researchers. Liu et al. [31] analyzed 11,748 articles related to Alzheimer’s disease and 693,938 references included in Web of Science from 2015 to 2019 and determined the research frontier and development trend by searching the keyword frequency. Gao et al. [32] used CiteSpace to visually analyze the literature related to CRISPR gene editing technology and concluded that Dudna Jia and Zhang Feng made outstanding contributions. The current hot spot is the application of CRISPR in animals and plants. Zhong et al. [33] analyzed the research hotspots and frontiers of exercise on the molecular mechanism of cancer through CiteSpace and concluded that the keyword ’Warburg effect ‘ranked first, with the highest citation outbreak. Inflammation, hepatocellular carcinoma, epithelial mesenchymal transformation, and adipose tissue are the current research hotspots. Liu et al. analyzed the history and current situation of rice research from 1985 to 2014 [34]. It was found that, before 2014, the number of publications in Asia grew rapidly, while the quality gap was large compared with that in the United States. The future research focus is the genetic analysis of agronomic traits. There are 2099 studies on the application of ecological models in eutrophication in the Web of Science. Hu [35] used CiteSpace to sort them out and found that, in recent years, the trend of close coupling between modeling and new experimental data acquisition has been found. This research provides a higher predictive ability for future ecological models. Microplastics have serious physical and chemical hazards in biological systems and humans. Yao et al. [36] systematically analyzed the relevant literature and found that the United States and Germany have more research on freshwater microplastics, and most of the pollution sources are concentrated in lakes and rivers. Che et al. [37] retrieved published papers on crisis and risk communication (CRCR) from Web of Science (1986–2020) and Scopus (1979–2020) and revealed the main research topics in the field of CRCR, emerging research hotspots in various periods, and turning points in overall development through CiteSpace’s two dimensions of co-author network and co-citation network.

To classify the knowledge subjects in the field of environmental safety management research, the visual analysis software VOSviewer was used to analyze the retrieval results, and the auxiliary software CiteSpace was used to grasp the international changes and development trends in the field of environmental safety management in recent years from multiple levels and perspectives.

The remainder of this paper is organized as follows. Section 2 introduces the technical route and methods. Section 3 discusses the international environmental safety management related literature in the journal publication, keywords, authors, scientific research institutions, countries, and other co-occurrence relations and future hot trends. Section 4 summarizes the discussion section and explains the research significance and shortcomings of this study.

## 2. Materials and Methods

Based on VOSviewer software and CiteSpace software, a visual quantitative analysis of the relevant literature documents of international environmental safety management from 1997 to 2021 was carried out and explored its research time zone and development trend. The visualization software developed by VOSviewer and CiteSpace, running in the Java environment, summarizes the evolution process of environmental safety management and explores its development hotspots and future trends by analyzing visual knowledge maps and keyword time zone maps and highlighting tables [38,39,40]. It can be imported into the pure text format of the Web of Science core collection. To facilitate the view of keyword clustering, this study imports the data in VOSviewer into Pajek software. After running in Pajek, the clustering graph is adjusted as needed to obtain a unified color clustering graph result for later analysis.

According to the co-occurrence data of authors, research institutions and countries in VOSviewer and the visual analysis of the literature results in Web of Science, Excel is used to draw relevant tables to analyze the cooperation of authors, research institutions, and countries. Different nodes in the VOSviewer network diagram represent elements, such as author cooperation nodes, cooperation institutions, related keywords, and countries/regions; if the frequency of research hotspots or keywords is hotter, the node range is larger [41]; the connection between nodes represents cooperation, co-occurrence, or co-citation [42]; clustering or year manifests itself in different colors of nodes and lines [43,44].

**Figure 1:**
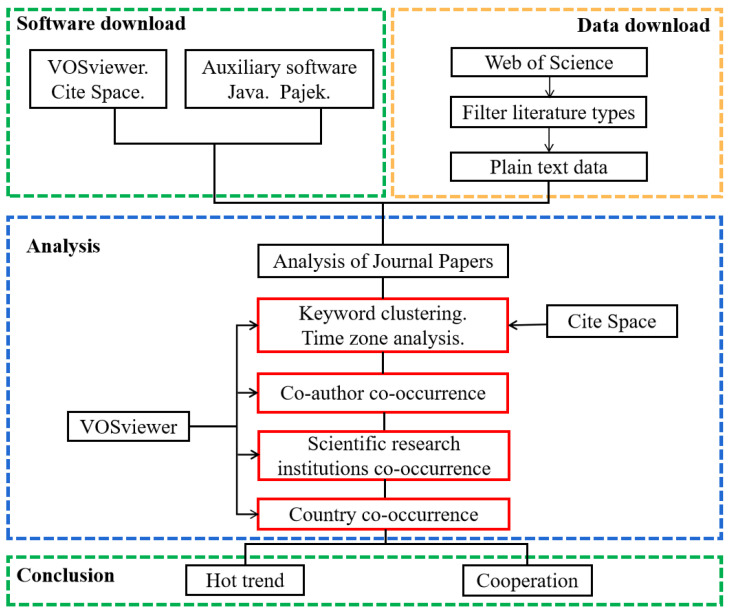
Technical roadmap.

The technical roadmap of this study is shown in Figure 1, which is mainly divided into four parts: visual analysis software download, data download, operation analysis, and result discussion [45].

### 2.1. Data Download

First, retrieval in the Web of Science core collection with Environmental safety management OR Environmental security management as the subject term was performed; the retrieval period was from 1 January 1997 to 31 December 2021; 7690 results were obtained. The literature types were selected as articles and review papers, and the final results numbered 7596. The search date is 22 March 2022. The content’s format is full-recorded and referenced references, with 500 exported plain text files per group.

### 2.2. Parameter Setting

Importing the above results obtained from the Web of Science literature to VOSviewer, the thresholds setting for exploring co-occurrences between keywords, authors, research institutions, and countries are as follows.

(1) Keyword setting. The minimum number of occurrences of a keyword is set to 5 in the VOSviewer software. Among the 31,117 keywords, 2161 keywords reached the threshold. Using its synonym dictionary to merge synonyms, such as climate change and climate-change, security, and safety, 2140 keywords reached the threshold, and their meaningless content words were removed at runtime. On the CiteSpace software, the data were first deduplicated, and then the date and project data were imported. The data time is set from January 1997 to December 2021, and the time slice setting parameter is two years.

(2) Author co-occurrence. In this study, the VOSviewer software parameter settings are as follows: the counting method is fractional counting, ignoring the literature research with more than 25 authors and setting the” Minimum number of authors per documents of an author” (the minimum number of documents per author) to 5 in 28,144 authors, and a total of 88 authors reached the threshold.

(3) Co-occurrence of research institutions. The minimum number of documents of one organization is set to 15, and 176 of the 7962 research institutions met the threshold.

(4) Co-occurrence of countries. The minimum number of documents of one country was set to 15. Among the 163 countries, there were 73 countries that reached the threshold.

## 3. Results

### 3.1. Journal Publication Analysis

According to what Web of Science retrieved in the “environmental safety management”-related literature, the annual publication statistics and the total proportion of papers on environmental safety management from 1997 to 2021 were collated from Web of Science.

**Figure 2:**
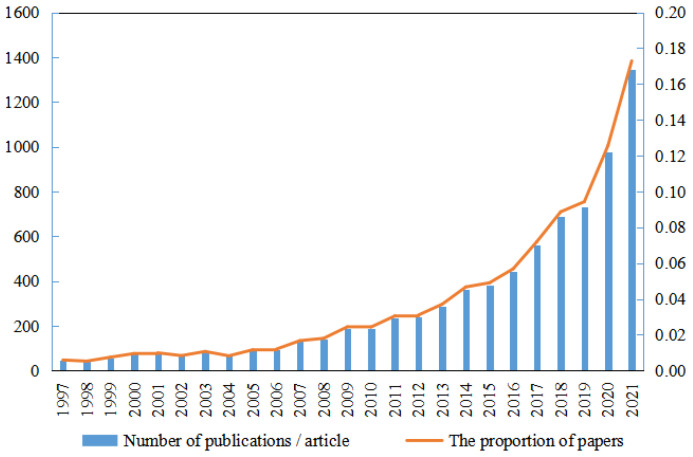
Statistics of annual documents issued by Web of Science on environmental safety management from 1997 to 2021.

As can be seen from Figure 2, the annual publication volume of the international literature on the theme of “environmental safety management” has shown a steady increase from 1997 to 2021. In 1997, the related topics of environmental safety management began to emerge, with only 46 articles. Along with time, the output results show an increasing trend. In 2017, the output of papers exceeded 500, and the largest number of papers was published in 2021, entering the peak period.

Within the collected 7596 data, the source of its journals is counted in the Web of Science, and the top 15 journals are sorted into Table 1.

In the data sample, according to the output of papers, the top five journals are “Sustainability”, “Journal of Cleaner Production”, “Science of The Total Environment”, “Journal of Environmental Management”, and “International Journal of Environmental Research and Public Health”. The top five journals are mostly environmental disciplines. The total number of output papers in the 15 journals reached 1445, accounting for 19.02 % of the total number of papers. In the cited influence, the top five highly cited journals in the past three years were “Plos One” (1,456,537), “Science of The Total Environment” (345,106), “Journal of Cleaner Production” (274,490), “Renewable & Sustainable Energy Reviews” (237,614), and “IEEE Access” (157,006).

According to the average IF, citation frequency and the number of articles published (referring to the number of papers or reviews on environmental safety management in its journals), “*Journal of Cleaner Production*” has the greatest influence, and the next are “*Science of The Total Environment*” and “*Renewable & Sustainable Energy Reviews*”. “*Journal of Cleaner Production*” mainly focuses on environmental science and ecology, environmental engineering, and green sustainable development technology. The major category of “*Science of The Total Environment*” is environmental science and ecology, which has certain authority and reliability in environmental safety management.

### 3.2. Keywords Heat and Cluster Analysis

Key words play an important role in the full-text focus and central research in a paper. The number of occurrences of key words is the total number of academic papers with the key words. The higher the frequency of keywords appearing in the paper, the more substantial the number of research results, the deeper and more concentrated the scope of research content. From the perspective of key words, the research hotspots and trends in this field can be reflected in a very comprehensive and high-level way [46]. In terms of the keyword heat and cluster analysis, VOSviewer is used for clustering and heat analysis; CiteSpace is used to analyze the time zone map and keyword emergence of keywords.

#### 3.2.1. Cluster Analysis of Keywords

**Figure 3:**
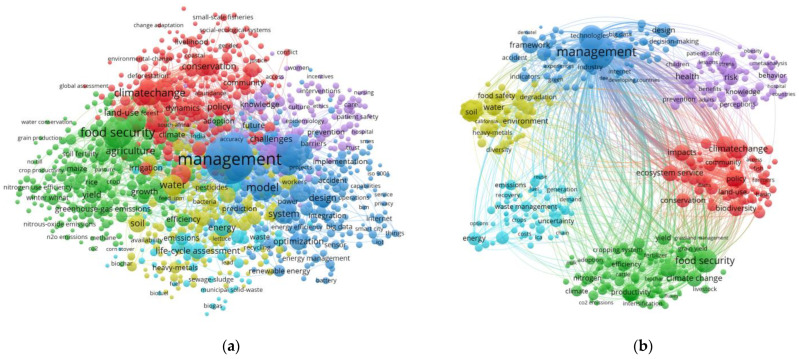
Keywords clustering map of environmental safety management from 1997 to 2021: (**a**) unadjusted; (**b**) after adjustment.

The literature retrieved from Web of Science was imported into VOSviewer1.6.9 software for extraction. After removing duplicate and meaningless keywords, cluster analysis was performed on keywords, and hotspot analysis was performed on high-frequency keywords. The software was run to draw the visual density map and cluster map. Figure 3a is the keyword clustering diagram. After the analysis of countries or keywords by VOSviewer, the relevant data can be downloaded in txt text and imported into Excel to count the word frequency, the number of nodes, and the total link strength. Due to the confusion of direct analysis, it is adjusted appropriately after running in Pajek and automatically imported into VOSviewer to obtain a monochromatic unified clustering map, as shown in Figure 3b.

It can be clearly seen from Figure 3b that keywords can be divided into six clusters. The main research directions can be summarized into the following six categories: (1) climate change, sustainable development, and biosafety management (red); (2) food production, agricultural pest management, and animal husbandry environmental safety management (green); (3) risk management, model optimization, and internet security (dark blue); (4) water security and ecological security management (yellow); (5) personal health and social safety management (purple), and (6) life cycle assessment, energy fuel, and waste management (light blue).

The results of cluster analysis show that the research hotspots of environmental security management in the world from 1997 to 2021 mainly focus on ecological security management, water management, climate change, agricultural food environmental security, social environmental management, and internet big data environmental security management. According to the keyword data downloaded by VOSviewer, the top 12 keywords are listed in Table 2. The research results of environmental safety management, such as food safety, sustainability, and climate change can be obtained.

**Figure 4:**
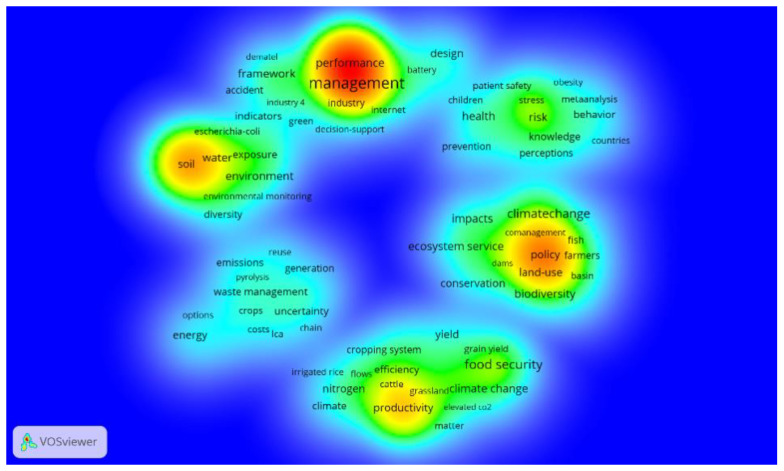
Keyword density visualization.

According to the density visualization diagram (Figure 4), the development hotspots of environmental safety management are explored. The dark color represents the high degree of heat and attention in this field. This study shows that there are many hot spots in the development of green sustainable development, food environmental safety, water safety, public environment, and other safety management. As for whether the global energy is effectively utilized, water resources, mineral resources, climate change, and other ecological environment security management issues are serious, which have attracted the attention and research of most scholars.

#### 3.2.2. Time Zone Diagram Analysis

**Figure 5:**
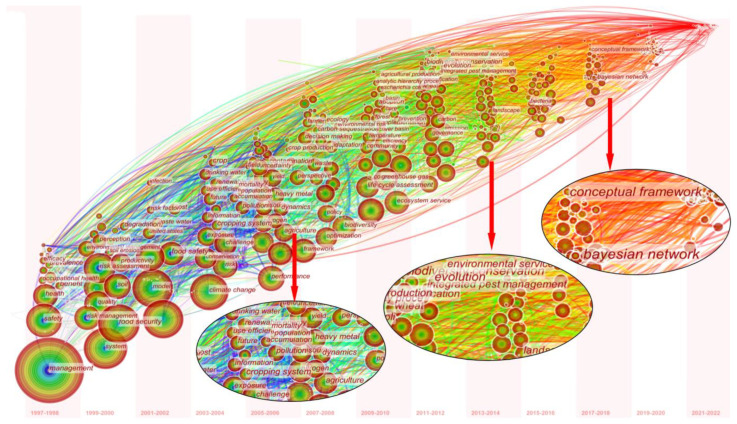
Time zone map of environmental safety management keywords from 1997 to 2021.

Using CiteSpace, the keyword time zone map and keyword burst map are obtained and shown in Figure 5. It can clearly understand when those keywords appear, so as to understand their hotspots and future trends.

With CiteSpace, the keyword time zone map is drawn and modified. The small circle is placed on the top, and the large circle is placed on the bottom and arranged in an orderly manner, which can more intuitively display the international time zone distribution of environmental safety management keywords. The larger the circle, the more times it is cited, and there are more discussions and research. There is a corresponding time below it. Taking two years as the time slice, the time distribution of each topic can be observed. Since the development of practical environmental safety management, such as social, agricultural ecological environment, and energy sustainable development, the safety management of the internet, such as Bayesian networks and conceptual frameworks, have gradually developed in recent years. The keyword emergence table is shown in Table 3, which can more intuitively show the trend development of related hotspots.

From the literature on environmental safety management, it can be seen that the word “safety” has appeared since 1997, which can be understood as the concept of environmental safety management in 1997. By the end of emergent in 2012, the overall correlation of safety is not very strong; Around 2000, the key words of risk management, risk assessment, degradation, injury, soil, and other environmental safety management related to risk assessment management, green sustainability, occupational safety, and agricultural soil began to emerge, attracting the attention and exploration of relevant scholars, with the correlation intensity as high as 16.27 and 17.32, and the end of the emergence being around 2010.

Around 2007, the themes of social safety management, such as markets, pest management and biofuels, agricultural pest management, and energy security, began to emerge, which means that international attention and research on social market safety management and agricultural pest control began to be paid attention to. With the development of economy and society, the global population reproduction mode is undergoing profound changes, which leads to great changes in global population development. The population base is now at the highest point, and food is a necessity for human beings. In the new era of rapid agricultural development, developing countries need not only to learn from the experience of agricultural safety management in developed countries and improve the ability of agricultural safety supervision, but also to analyze the problems and corresponding countermeasures in agricultural environmental safety management [47]. In recent years, hot words such as big data, internet, smart city, artificial intelligence, and other security management in the internet environment have begun to emerge and continue to this day. In case of information leakage and core technology leakage, it will cause extremely serious impact and consequences on society and factories. From travel payment to education business, internet environment security management must strengthen the response and management to fully guarantee internet security [48].

Whether it is social ecological environment or network virtual environment, its security management is worth exploring and improving. Analyzing the problems existing in environmental management so as to improve and optimize the relevant deficiencies in laws and regulations, relevant regulatory authorities, and future education and training is important.

### 3.3. Cluster Analysis of Authors, Research Institutions and Countries

#### 3.3.1. Cooperation among Authors

**Figure 6:**
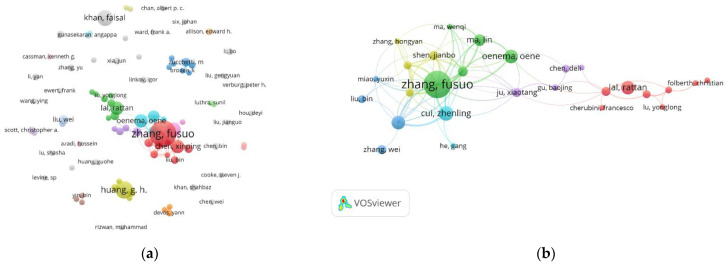
(**a**) Co-occurrence graph of author collaboration; (**b**) co-occurrence diagram of author collaboration (small group of 27 people).

**Figure 7:**
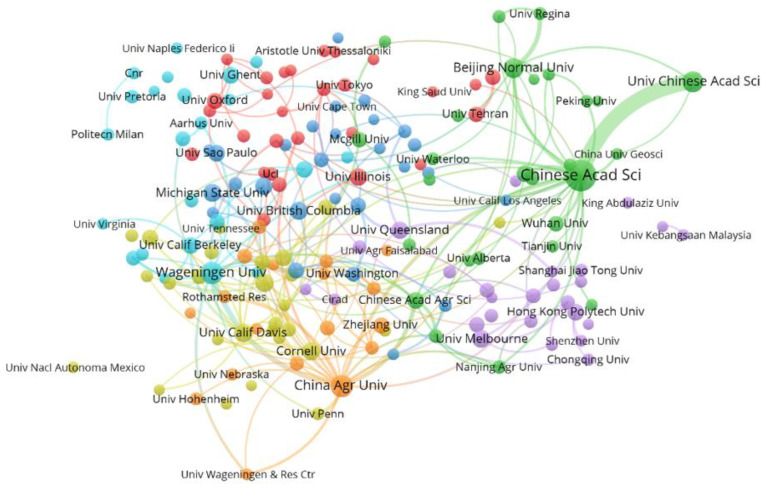
Co-occurrence of research institutions on environmental safety management from 1997 to 2021.

The existence of excellent scholars in the research field is the key factor for the development of this field, and the scholars in the research field are the important force to promote the development of this field. In VOSviewer, 28,144 authors were obtained by running the data, and 88 results were obtained by running the software for authors with more than five papers. Some of the 88 projects in the network are not connected to each other (as shown in Figure 6). The largest group of links consists of 27 projects (as shown in Figure 7), showing that 27 authors have formed six collaborating groups. The largest number of group members is eight. There are four members in the three groups, and five and two members are in the other two groups. In the author’s cooperative co-occurrence clustering diagram, it was found that the research group centered on Zhang Fusuo, a scholar from China Agricultural University, who was the largest contributor.

According to the cluster analysis of keywords, environmental safety management is divided into different disciplines, such as agricultural ecological environment, social environment, water environment, energy environment, and computer network environment. There is a certain relationship between these disciplines, such as food environmental safety management and computer environmental safety management. The general direction is safety management. The management details will also be different, so there are few or even no links between the groups. Only a few scholars have established links, and the cooperation between the members of the groups is not close. Some of the authors of the connection groups cooperate closely and widely, and some research fields are mainly independent research. Most of the authors of the largest groups focus on ecological environment, agriculture, urban pollution, etc. The other authors do not cooperate closely. The relevant information of the authors with more than 10 articles is shown in Table 4.

According to the author’s data, the top three authors who published the most papers are FuSuo, Zhang (China Agricultural University), GuoHe, Huang (Beijing Normal University), and Faisal I, Khan (Memorial University of Newfoundland). FuSuo, Zhang [49] studies the basic science of crops and agriculture, GuoHe, Huang studies environmental science and resource utilization, and Faisal I, Khan [50] studies process control and simulation. The link strength between Faisal I, Khan and the other authors is 0 because environmental safety management is the safety management of different disciplines, mainly involving small-scale group cooperation.

#### 3.3.2. Cooperation among International Research Institutions

The output of international environmental safety management research from 1997 to 2021 is shown in Figure 7. According to the connection between institutions, the Chinese Academy of Sciences and the University of Chinese Academy of Sciences cooperate closely. Additionally, the cooperation between scientific research institutions in various countries is relatively close.

The visual analysis results of inter-agency cooperation obtained by VOSviewer software are shown in Table 5. The Chinese Academy of Sciences has published 224 papers, ranking first. The other top 15 high-yield institutions were China Agricultural University (91), Wageningen University (89), University of Chinese Academy of Sciences (79), Beijing Normal University (65), University of California Davis (58), University of Florida (54), University of British Columbia (52), Cornell University (51), University of Melbourne (51), Michigan State University (49), University of Queensland (48), Ohio State University (46), University of Illinois (45), and Tsinghua University (44). Among the top 15 institutions in the number of papers published from 1997 to 2021, there are five institutions from China, reflecting that Chinese research institutions are becoming more and more active in international environmental security management. In addition, a high-level research team and cutting-edge research fields have been formed, which have made important contributions to the development of international environmental safety management.

The results shown in Table 6 are obtained by ranking all institutions according to the number of citations. The most cited are the University of Minnesota and the University of California, Santa Barbara. The University of Minnesota is one of the most comprehensive institutions of higher learning in the United States. The University of California, Santa Barbara, is one of the world’s leading public research universities, especially in science and technology and media. The top five institutions cited were the University of Minnesota, the University of California, Santa Barbara, the Chinese Academy of Sciences, McGill University, and China Agricultural University. Although Chinese scientific research institutions have issued more papers, accounting for 48.3% of the top 15 rankings, only three universities have appeared in the top 15 rankings of citations, and the number of citations is not high. The results indicate that the status of relevant scientific research institutions in China is not very authoritative and still needs to continue to develop.

#### 3.3.3. Cooperation between International Countries

**Figure 8:**
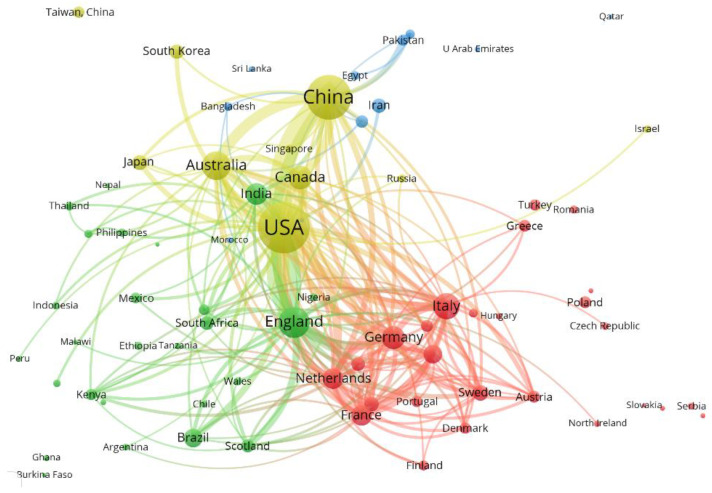
Cooperation among Environmental Security Management Countries.

When analyzing the knowledge subjects in the field of environmental safety management, the country is taken as the analysis unit [51]. In VOSviewer, select ”Country” as the research node to obtain the national (regional) cooperation knowledge map. The results show that as shown in Figure 8, there are 73 nodes, and one country (region) is represented by a node. The color of the node reflects the time and number of documents issued by different countries, and the size of the node reflects the frequency of documents issued by different countries. The largest node in the figure is the United States in yellow, indicating that the United States has a dominant authority in the subject area. This is followed by China, Australia, and the United Kingdom, and the number and frequency of publications are also higher. According to the thickness of the connection between countries, it can be seen that China has close cooperation with the United States, Britain, Australia, and India, and Chinas has relatively weak cooperation with Italy, France, Japan, and Germany in scientific research. The United States has close cooperation with China, Britain, Italy, Germany, and France.

According to the issuance of documents, the ranking of environmental safety management is shown in Table 7. The United States ranks first in publication, citation frequency, and total link strength; China ranks second in terms of publication and citation, but the overall link strength is slightly lower than that of the United Kingdom, indicating that China is not very close to some countries in scientific research cooperation.

## 4. Conclusions

(1) Bibliometric results show that environmental safety management papers have been published year by year since 1997, and the number of published papers has increased rapidly year by year. According to the average IF, citation frequency, and number of publications, the most influential is “*Journal of Cleaner Production*”, followed by “*Science of The Total Environment*” and “*Renewable & Sustainable Energy Reviews*”.

(2) VOSviewer’s keyword co-occurrence map analysis yielded six clusters: (a) climate change, sustainable development, and biosafety management (red); (b) food production, agricultural pest management, and animal husbandry environmental safety management (green); (c) risk management, model optimization, and internet security (dark blue); (d) water security and ecological security management (yellow); (e) personal health and social safety management (purple), and (f) life cycle assessment, energy fuel, and waste management (light blue). CiteSpace’s keyword time zone map and burst analysis show that the focus of international environmental security management research has shifted from social, agricultural, and ecological environments to the security management of internet environments, such as Bayesian networks and conceptual frameworks.

(3) Due to environmental safety management classification, differences between disciplines, cooperation between authors is not close. The largest group cooperation is composed of 27 scholars, with FuSuo, Zhang of China Agricultural University as the center, and their main research field is crop and agricultural basic science. This is followed by GuoHe, Huang, a scholar of Beijing Normal University, whose research field is environmental science and resource utilization.

(4) The cooperation between scientific research institutions in various countries is relatively close. The cooperation between the Chinese Academy of Sciences and the University of Chinese Academy of Sciences is close. Chinese scientific research institutions are increasingly active in international environmental safety management and have formed a high-level research team and frontier research fields. However, the citations of Chinese scientific research institutions are relatively few.

(5) The United States currently occupies a dominant and authoritative position in this field, followed by China, the United Kingdom, and Australia. China has closer cooperation with the United States, Britain, Australia, and India; the United States has close cooperation with China, Britain, Italy, Germany, and France.

In the past, most of the research directions in the field of environmental safety management were distributed in building environmental safety, coal mine environmental safety, and agricultural ecological environment safety. At present, the real practical concerns in the field of environmental safety management are mainly concentrated in the fields of society, energy, and ecological construction under the internet. These are represented by intelligent fire protection, which combines the fire protection field with the internet field, from a single environmental safety management to a diversified and intelligent direction. This study uses visualization software to cluster keywords and analyze time zones. The forthcoming hot trends in the field of environmental safety management have been identified. According to the analysis results, the forthcoming hot trends in the field of environmental security management have gradually changed from ecological environment security, social environment security, energy environment security, and urban environment security to network environment security. Therefore, the results of this analysis are consistent with the real practical concerns in the field of environmental safety management. At the same time, the results of this study provide guidance and suggestions for researchers around the world. In other words, this opens a new door for them to help more researchers conduct more in-depth research in the field of network environment security management. In the 21st century, these trends are more rapidly developing in the network era. According to the results of visual analysis, the current research hotspots on environmental safety management are gradually changing, but this does not mean that the previous research categories are no longer valued. Throughout the research and analysis of environmental security management from 1997 to 2021, it can be seen that environmental security management has traditional and non-traditional hot spots. Traditional research hotspots refer to environmental security management in the actual environment, while non-traditional research indicates the internet environment security that has been paid more attention to in recent years. With the advent of the internet era, information has become digital, rapid, global, and diversified. The combination of traditional and non-traditional fields can make the work of environmental safety management more efficient, such as smart city and smart fire protection. Making good use of data analysis will bring brainstorming ideas to future research directions. VOSviewer and CiteSpace play an indispensable role in the future research trend of environmental security.

Compared with other similar reviews, for the first time, this study analyzes the international and domestic environmental safety management field through VOSviewer and CiteSpace visualization software. This study analyzes the clustering, density, time zone distribution, and highlights of keywords. The results show that the current environmental safety management field is widely involved. These fields mainly include ecological environment security management, social environment security management, energy environment security management, urban environment security management, and network environment security management. Moreover, the research hotspots in the field of environmental security management have changed, which has been reflected in the environmental security management of internet big data. Before this, few researchers used visualization softwares, such as VOSviewer, to analyze the field of environmental safety management. So, this is a new attempt. Based on the literature source of Web of Science database, this study uses visual analysis software to sort out the research literature of environmental safety management, analyze the hot topics and evolution paths of environmental safety management research, and explore the characteristics and future development trends of environmental safety management research. This study is conducive to building a theoretical library of environmental safety management research and provides theoretical reference for scholars to analyze the research status of environmental safety management and open new research frontiers.

This study uses VOSviewer and Cite Space to analyze the big data of environmental safety management in the core collection of Web of Science, and this study can efficiently and clearly obtain visual maps of keywords, co-authors, scientific research institutes, countries, etc. On this basis, the current research hotspots and future trends in this field have received sufficient attention. At the same time, the relevant literature of interest in this field can also be targeted learning. Moreover, this method is especially suitable for beginners. For example, for a first-year graduate student, when he does not know where to start in a large research field, using visual analysis software to analyze big data can obtain some valuable inspiration. However, this paper also has some shortcomings. Although Web of Science is one of the largest databases at present, it does not mean that the data here is very sufficient. Data information in these databases, such as Scopus and Elsevier Science, can also be effectively utilized. When the information in multiple databases is gathered, the latest and cutting-edge hot information will be mined. Grasping those diversified and potential research hotspots will bring new ideas and scientific research directions to the research in this field.

## Figures and Tables

**Table 1:** Information about the top 15 journals in the output of environmental safety management papers.

Number	Journal Name	JCR Partition	IF	Citation Frequency	Number of Publications	%/7596
1	Sustainability	Q2	3.889	106,733	299	3.94%
2	Journal of cleaner production	Q1	11.072	274,490	238	3.13%
3	Science of the total environment	Q1	10.753	345,106	146	1.92%
4	Journal of environmental management	Q1	8.910	107,102	105	1.38%
5	International journal of environmental Research and public health	Q1	4.614	98,039	96	1.26%
6	Environmental science and pollution Research	Q2	5.190	118,794	82	1.08%
7	Water	Q2	3.530	38,831	71	0.93%
8	Safety science	Q1	6.392	29,814	64	0.84%
9	Plos one	Q2	3.752	1,456,537	63	0.83%
10	Energies	Q3	3.252	78,460	51	0.67%
11	Renewable and sustainable energy reviews	Q1	16.799	237,614	51	0.67%
12	IEEE access	Q2	3.476	157,006	46	0.61%
13	Ocean and coastal management	Q1	4.295	16,688	46	0.61%
14	Agricultural water management	Q1	6.611	39,269	45	0.59%
15	Agricultural systems	Q1	6.765	16,695	42	0.55%
Sum	-	-	-	-	1445	19.02%

**Table 2:** Keyword frequency sorting.

Keyword	Word Frequency	Total Correlation Strength
Management	1553	9032
Safety	691	3587
Food security	590	4348
Climate change	590	4113
Systems	519	3222
Impact	506	3359
Sustainability	438	2857
Model	379	2080
Performance	251	1413
Water	248	1518
Agriculture	242	1842
Framework	238	1544

**Table 3:** Key words of environmental safety management 1997–2021.

Keywords	Strength	Begin	End	1997–2021
Safety	8.86	1997	2012	▃▃▃▃▃▃▃▃▃▃▃▃▃▃▃▃ ▂▂▂▂▂▂▂▂▂
Prevalence	7.44	1997	2012	▃▃▃▃▃▃▃▃▃▃▃▃▃▃▃▃ ▂▂▂▂▂▂▂▂▂
Risk management	17.32	1999	2010	▂▂ ▃▃▃▃▃▃▃▃▃▃▃▃ ▂▂▂▂▂▂▂▂▂▂▂
Risk assessment	16.27	1999	2010	▂▂ ▃▃▃▃▃▃▃▃▃▃▃▃ ▂▂▂▂▂▂▂▂▂▂▂
Degradation	6.56	1999	2010	▂▂ ▃▃▃▃▃▃▃▃▃▃▃▃ ▂▂▂▂▂▂▂▂▂▂▂
Injury	6.24	1999	2014	▂▂ ▃▃▃▃▃▃▃▃▃▃▃▃▃▃▃▃ ▂▂▂▂▂▂▂
Soil erosion	5.4	1999	2012	▂▂ ▃▃▃▃▃▃▃▃▃▃▃▃▃▃ ▂▂▂▂▂▂▂▂▂
Food safety	15.23	2001	2012	▂▂▂▂ ▃▃▃▃▃▃▃▃▃▃▃▃ ▂▂▂▂▂▂▂▂▂
Risk factor	5.64	2001	2012	▂▂▂▂ ▃▃▃▃▃▃▃▃▃▃▃▃ ▂▂▂▂▂▂▂▂▂
Quality	10.05	2003	2014	▂▂▂▂▂▂ ▃▃▃▃▃▃▃▃▃▃▃▃ ▂▂▂▂▂▂▂
Exposure	7.34	2003	2010	▂▂▂▂▂▂ ▃▃▃▃▃▃▃▃ ▂▂▂▂▂▂▂▂▂▂▂
Double blind	7.03	2003	2012	▂▂▂▂▂▂ ▃▃▃▃▃▃▃▃▃▃ ▂▂▂▂▂▂▂▂▂
Mortality	5.33	2003	2014	▂▂▂▂▂▂ ▃▃▃▃▃▃▃▃▃▃▃▃ ▂▂▂▂▂▂▂
Population	5.08	2003	2014	▂▂▂▂▂▂ ▃▃▃▃▃▃▃▃▃▃▃▃ ▂▂▂▂▂▂▂
Environmental health and safety	4.99	2003	2010	▂▂▂▂▂▂ ▃▃▃▃▃▃▃▃ ▂▂▂▂▂▂▂▂▂▂▂
Resistance	6.47	2007	2012	▂▂▂▂▂▂▂▂▂▂ ▃▃▃▃▃▃ ▂▂▂▂▂▂▂▂▂
Science	5.19	2007	2014	▂▂▂▂▂▂▂▂▂▂ ▃▃▃▃▃▃▃▃ ▂▂▂▂▂▂▂
Diofuel	9.28	2009	2014	▂▂▂▂▂▂▂▂▂▂▂▂ ▃▃▃▃▃▃ ▂▂▂▂▂▂▂
Health	6.42	2009	2014	▂▂▂▂▂▂▂▂▂▂▂▂ ▃▃▃▃▃▃ ▂▂▂▂▂▂▂
Market	5.87	2009	2016	▂▂▂▂▂▂▂▂▂▂▂▂ ▃▃▃▃▃▃▃▃ ▂▂▂▂▂
Agricultural intensification	6.82	2011	2016	▂▂▂▂▂▂▂▂▂▂▂▂▂▂ ▃▃▃▃▃▃ ▂▂▂▂▂
Ecosystem	5.75	2015	2018	▂▂▂▂▂▂▂▂▂▂▂▂▂▂▂▂▂▂ ▃▃▃▃ ▂▂▂
Resource management	5.08	2015	2018	▂▂▂▂▂▂▂▂▂▂▂▂▂▂▂▂▂▂ ▃▃▃▃ ▂▂▂
Matter	5.59	2017	2018	▂▂▂▂▂▂▂▂▂▂▂▂▂▂▂▂▂▂▂▂ ▃▃ ▂▂▂
Big data	5.47	2017	2021	▂▂▂▂▂▂▂▂▂▂▂▂▂▂▂▂▂▂▂▂ ▃▃▃▃▃
Internet	10.08	2019	2021	▂▂▂▂▂▂▂▂▂▂▂▂▂▂▂▂▂▂▂▂▂▂ ▃▃▃
Smart city	6.62	2019	2021	▂▂▂▂▂▂▂▂▂▂▂▂▂▂▂▂▂▂▂▂▂▂ ▃▃▃
Barrier	6.24	2019	2021	▂▂▂▂▂▂▂▂▂▂▂▂▂▂▂▂▂▂▂▂▂▂ ▃▃▃
Surface water	6.09	2019	2021	▂▂▂▂▂▂▂▂▂▂▂▂▂▂▂▂▂▂▂▂▂▂ ▃▃▃
Thing	5.3	2019	2021	▂▂▂▂▂▂▂▂▂▂▂▂▂▂▂▂▂▂▂▂▂▂ ▃▃▃

**Table 4:** Author publication statistics.

Author	Number of Publications (*n*)	Total Connection Strength
Zhang, Fusuo	29	73
Huang, Guohe	18	18
Khan, Faisal I	16	0
Cui, Zhenling	14	39
Chen, Xinping	13	34
Lal, Rattan	13	2
Oenema, Oene	13	22
Ma, Lin	11	25

**Table 5:** Ranking of research institutions by papers’ number.

Organization	Documents	Number of Citations	Total Connection Strength
Chinese Acad Sci	224	6860	213
China Agr Univ	91	5259	110
Wageningen Univ	89	3927	109
Univ Chinese Acad Sci	79	2028	108
Beijing Normal Univ	65	1783	59
Univ Calif Davis	58	3334	74
Univ Florida	54	1812	80
Univ British Columbia	52	1095	48
Cornell Univ	51	2154	44
Univ Melbourne	51	1936	35
Michigan State Univ	49	2888	69
Univ Queensland	48	1702	60
Ohio State Univ	46	2045	28
Univ Illinois	45	1353	55
Tsinghua Univ	44	3533	29

**Table 6:** Ranking of research institutions by number of citations.

Organization	Documents	Number of Citations	Total Connection Strength
Univ Minnesota	36	10,292	55
Univ Calif Santa Barbara	24	8657	33
Chinese Acad Sci	224	6860	213
Mcgill Univ	36	6774	55
China Agr Univ	91	5259	110
Stockholm Univ	25	4981	71
Arizona State Univ	31	4972	43
Univ Wisconsin	40	4833	54
Univ Bonn	28	4795	29
Wageningen Univ	89	3927	109
Stanford Univ	30	3846	51
Tsinghua Univ	44	3533	29
Univ Calif Davis	58	3334	74
Michigan State Univ	49	2888	69
Univ Penn	22	2780	32

**Table 7:** Cooperation between countries and total link strength.

Country	Documents	Citation	Total Connection Strength
USA	1809	76,651	1625
China	1346	37,569	1014
UK	671	25,128	1187
Australia	560	20,847	782
Italy	475	13,709	768
Canada	403	19,226	609
Germany	378	16,488	779
India	332	10,026	425
Netherlands	305	12,838	685
France	300	12,679	656

## Data Availability

The data presented in this study are available upon request from the corresponding author.
